# Associations between lifetime potentially traumatic events and chronic physical conditions in the South African Stress and Health Survey: a cross-sectional study

**DOI:** 10.1186/s12888-016-0929-z

**Published:** 2016-07-07

**Authors:** Lukoye Atwoli, Jonathan M. Platt, Archana Basu, David R. Williams, Dan J. Stein, Karestan C. Koenen

**Affiliations:** Department of Mental Health, Moi University School of Medicine, PO Box 1493, Eldoret, 30100 Kenya; Department of Psychiatry and Mental Health, MRC Unit on Anxiety and Stress Disorders, University of Cape Town, Cape Town, South Africa; Department of Epidemiology, Mailman School of Public Health, Columbia University, New York, NY USA; Department of Epidemiology, Harvard T.H. Chan School of Public Health, Boston, MA USA; Department of Social and Behavioral Sciences, Harvard T.H. Chan School of Public Health, Boston, MA USA; Psychiatric and Neurodevelopmental Genetics Unit, Center for Human Genetic Research, Massachusetts General Hospital, Boston, MA USA

**Keywords:** Trauma, Physical health, South Africa, Posttraumatic stress disorder

## Abstract

**Background:**

This study examined the association between the type, and cumulative number of lifetime potentially traumatic events (PTEs), and chronic physical conditions, in a South African sample. PTE exposures have been associated with an increased risk for a wide range of chronic physical conditions, but it is unclear whether psychiatric disorders mediate this association. Given the established differences in trauma occurrence, and the epidemiology of posttraumatic stress disorder (PTSD) in South Africa relative to other countries, examining associations between PTEs and chronic physical conditions, particularly while accounting for psychiatric comorbidity is important.

**Methods:**

Data were drawn from the South African Stress and Health Study, a cross-sectional population-representative study of psychological and physical health of South African adults. Twenty-seven PTEs, based on the World Health Organization Composite International Diagnostic Interview Version 3.0, DSM-IV PTSD module were grouped into seven PTE types (war events, physical violence, sexual violence, accidents, unexpected death of a loved one, network events, and witnessing PTEs). Five clusters of physical conditions (cardiovascular, arthritis, respiratory, chronic pain, and other health conditions) were examined. Logistic regressions assessed the odds of reporting a physical condition in relation to type and cumulative number of PTEs. Cochran-Armitage test for trend was used to examine dose-response effect of cumulative PTEs on physical conditions.

**Results:**

After adjusting for sociodemographic variables and psychiatric disorders, respondents with any PTE had increased odds of all assessed physical conditions, ranging between 1.48 (95 % CI: 1.06–2.07) for arthritis and 2.07 (95 % CI: 1.57–2.73) for respiratory conditions, compared to those without PTE exposure. Sexual violence, physical violence, unexpected death of a loved one, and network PTEs significantly increased the odds of all or nearly all the physical conditions assessed. There was a dose-response relationship between number of PTEs and increased odds of all physical conditions.

**Conclusions:**

Results from this study, the first in an African general population, are consistent with other population-based studies; PTEs confer a broad-spectrum risk for chronic physical conditions, independent of psychiatric disorders. These risks increase with each cumulative PTE exposure. Clinically, comprehensive evaluations for risk of mental and physical health morbidities should be considered among PTE survivors.

## Background

Exposure to potentially traumatic events (PTE) has been associated with a range of physical health problems (eg [1–4]) with effect sizes similar to PTE-consequent mental health outcomes [5, 6]. This presents a significant public health concern given the rates of PTE occurrence and posttraumatic stress disorder (PTSD) prevalence in South Africa [7], and the high and rising burden of non-communicable diseases in Africa [8]. Thus, examining the link between PTEs, their mental health sequelae, and chronic physical conditions is important.

PTEs have been associated with a wide range of biomarkers (eg, immune system inflammation, cortisol response) representing mechanisms believed to underlie health problems [9]. Several disease states including cardiovascular disease [3, 10–12], chronic pain [13–16] and headaches [17], arthritis and other autoimmune diseases [4, 18–20], pulmonary problems [3, 21, 22], Type II diabetes [23–26], digestive problems [26], and even disease mortality [27, 28] have been related to PTEs. Studies examining violence exposure-witnessing or experiencing-including intimate partner violence, physical and/or sexual abuse [10, 29–31], as well as motor vehicle accidents [32], and combat exposure [12, 14, 27], among other trauma types, suggest that exposure to PTEs and/or developing PTSD are risk factors for physical health conditions.

Despite this burgeoning literature, some key issues remain under examined. First, there is debate as to whether PTE exposure itself is a sufficient risk factor for poor health, or whether PTE sequelae, particularly PTSD, are necessary factors [33–35] for chronic physical health problems. Relatedly, while several studies have shown a dose-response between exposure to number of PTEs and increased risk of physical health problems (eg [1, 17, 19, 36]), some reports suggest that cumulative PTE exposure may be confounded with PTSD [37–39]. Unraveling these issues has important implications for public health policy, since prevalence rates of PTE exposure are significantly higher than PTSD [40]. Accordingly, this also has implications for how intervention efforts are targeted-will treating PTSD also address the risk for poor physical health sequelae, or is a more comprehensive preventive approach to trauma exposure warranted? Thus far, when compared to studies focused on specific types of trauma, findings from population-based studies that examine lifetime cumulative PTE exposure, including samples from the United States [19] and cross-national data [1, 41], suggest that exposure to PTEs and PTSD are independently associated with physical health outcomes. Based on these findings, this study sought to investigate the association between both the type and cumulative number of lifetime PTEs, and chronic physical conditions. Relatedly, this study also aims to provide evidence to guide interventions designed to address PTE sequelae and chronic physical conditions, particularly in South Africa. Second, it is notable that most studies examining the impact of PTEs on physical health have been carried out in the U.S. and other relatively high-income countries. There is some evidence to suggest that these associations may vary by country [1]. Additionally, previous work on PTEs and PTSD in South Africa has shown that these distributions differ significantly from those seen in more developed countries [7]. Therefore, this study also seeks to highlight areas of future inquiry to understand the impact of PTEs in South Africa.

## Methods

### Data source and study population

Data were drawn from the South Africa Stress and Health Study (SASH), the first survey of physical and psychological trauma and concomitant disorders among a population-representative sample of South African adults ages 18 and older [42]. The cross-sectional survey was administered between January 2002 and June 2005, using a fully structured pencil and paper questionnaire in one of the six major languages in South Africa. Human subjects committees of the University of Michigan, Harvard Medical School, and the Medical University of Southern Africa approved all study recruitment, consent, and field procedures. The study sample was created using a multistage, area probability sample of civilian non-institutionalized adults living in households and group hostels, identified through use of 2001 South African Census of Enumerations Areas. Detailed study rationale and methodology has been previously published [42]. A total of 5000 individuals were initially selected, and 4433 (87.1 %) were interviewed. Due to various exclusion criteria, the final study sample included 4351 (85.5 %) individuals. Final survey data were weighted to adjust for sample clustering, participant selection probabilities, and non-response rates. Additionally, data were weighted to reflect the demographic frequencies of gender, age, race, and geography according to the 2001 South Africa Census [43].

## Measures

### Potentially traumatic events

A total of 27 PTEs were included as separate exposures. Measures were taken from the World Health Organization (WHO) Composite International Diagnostic Interview (CIDI) Version 3.0, Diagnostic and Statistical Manual of Mental Disorders-IV (DSM-IV) [44] PTSD module, a fully structured pencil and paper questionnaire for use by interviewers without any clinical experience [45]. Lifetime exposure to 27 PTEs were categorized into seven types: war events (combat, relief worker in a war zone, civilian in a war zone, civilian in a region of terror, refugee and purposely injured, tortured or killed someone), physical violence (physical abuse by caregiver, physical assault by spouse or romantic partner, physical assault by someone else, mugged or threatened with a weapon, and kidnapped), sexual violence (raped, sexually assaulted, and stalked), accidents (toxic chemical exposure, automobile accident, natural disaster, man-made disaster, and other life-threatening accident), unexpected death of a loved one, network events involving others (having a child with a serious illness, traumatic event occurring to a loved one, and accidentally causing serious injury or death), and witnessing (witnessing a death, seeing a dead body or someone seriously hurt, seeing atrocities, and witnessing domestic violence). The questionnaire also asked respondents to report private/other PTEs, which were not included in these analyses due to inadequate statistical power. Complete sample characteristics of PTEs have been previously published [7]. Because the outcomes of interest were physical conditions, life-threatening illness was not included as a PTE. A variable representing the total number of PTEs was also created by summing the number of events an individual reported having experienced (from 0 to 27). The SASH did not capture the number of times any one PTE was experienced, so PTEs were counted only once for each reported type. PTE types were examined as dichotomous variables (yes/no), and as a cumulative exposure (0–27).

### Physical health

Physical health outcomes were queried for 20 conditions using a dichotomous self-report module for previous year incidence. The conditions were then aggregated by type in order to maintain adequate statistical power. There were five final categories: cardiovascular disease (heart disease, high blood pressure, stroke, heart attack), arthritis, respiratory conditions (asthma, other chronic lung disease, seasonal allergies), chronic pain (chronic back or neck problems, frequent/severe headaches, other chronic pain), and other conditions (tuberculosis, malaria, diabetes, ulcer, thyroid, neurological conditions, HIV/AIDS, epilepsy, cancer). An additional variable was created to represent any previous year physical condition (yes/no). Self-report modules of this type have shown good reliability [46, 47] and fair validity [48].

### Mental and substance use disorders

Mental health symptoms were collected using the CIDI 3.0 tool, in order to determine lifetime mental disorder prevalence. Diagnoses were based on DSM-IV criteria. The mental disorders included in this study are mood disorders, anxiety disorders, and substance use disorders. PTSD, based on the WHO CIDI scale of 27 PTEs, was analyzed separately from other anxiety disorders.

### Demographic covariates

Six sociodemographic variables were included in final study models, including sex, age (18–29, 30–44, 45–59, 60+ years), marital status (married, previously married and never married), education (low (0–1 years), low-average (2–7 years), high-average (8–12 years) and high (13+ years), employment status (employed, homemaker, retired and other (including unemployed and students), and race (Black, White, Indian/Asian, and Colored, a historical South African classification for a heterogeneous racial group from mixed ancestry) [49]. The sociodemographic variables age and education were grouped categorically and were analyzed using dummy variables. Variables were selected and organized according to a previously published article on PTEs and PTSD epidemiology in the SASH sample [7].

## Analysis

### Frequency testing

The study examined the association between both type and number of cumulative lifetime PTEs and previous year physical health conditions. To accurately capture the association of interest, only individuals who reported PTEs prior to the onset of any physical condition were included. Rao-Scott chi-square tests were used to determine whether demographic groups reported statistically significant differences in the frequency of previous year physical conditions, as well as the number of cumulative reported PTEs. The Cochran-Armitage test for trend was used to examine whether the binomial proportions of physical conditions were statistically significantly different with each cumulative PTE [50].

### Logistic regression modeling

The odds of reporting a physical condition were tested among respondents who reported each type and cumulative number of PTEs using logistic regression modeling. Final models were adjusted for sex, age, education, employment, marital status, race/ethnicity, and any mood, anxiety, or substance use disorder, and PTSD. All tests were completed in STATA using weighted analysis as described in the study population description [51].

## Results

### Prevalence of physical conditions and potentially traumatic events (PTEs)

See Table 1 for full results. The most commonly reported physical condition was chronic pain (46.6 %). Up to 60.2 % of the sample reported having at least one physical condition. Female respondents reported a higher prevalence of all conditions that was statistically significant in all cases except respiratory conditions. Groups with the highest prevalence of physical conditions were female, age 60+, previously married, highly educated (13+ yrs), and worked as a homemaker. Further, prevalence of mental disorders among those who reported any physical condition ranged from 61.2 % for substance use disorders to 93.4 % for PTSD, evidence of high comorbidity between lifetime mental disorders and physical conditions. The most commonly reported PTE types were physical violence (32.9 %), sexual violence (28.4 %), and unexpected death of a loved one (31.3 %). Overall, the frequencies of any reported physical condition were elevated among respondents who experienced all PTE types, ranging from 64.7 % (witnessing) to 79.2 % (network trauma).

**Table 1:** Prevalence of physical conditions among sociodemographic groups and PTE types (*n* = 4351)

		Type of physical condition (%)^a^					
	Total sample	Arthritis	Cardio-vascular	Respiratory	Chronic pain	Other Condition	Any physical condition
Total (n; %)	4351 (100 %)	455 (10 %)	915 (19.5 %)	843 (19.1 %)	2055 (46.6 %)	729 (16.5 %)	2656 (60.2 %)
Sex							
Female	53.6	13.7	25.9	19.6	53.6	18	66.8
Male	46.4	5.8	12.2	18.6	38.5	14.9	52.6
*p*-value*		<.0001	<.0001	0.434	<.0001	0.009	<.0001
Age							
18–29	39.1	2.4	5.7	17.8	38.9	9.3	49.1
30–44	32.1	7.1	15.4	19.1	46.4	16.6	59.8
45–59	20	19.2	37.7	20.5	54.2	24.5	72.2
60+	8.7	33.6	55.2	22.7	64.1	29.5	83.3
*p*-value*		<.0001	<.0001	0.193	<.0001	<.0001	<.0001
Marital Status							
Currently married	50.6	13	25	20.2	51.6	19.4	64.8
Previously married	6.5	19.7	39.2	15.6	55.1	20.3	76.6
Never Married	42.9	5.2	10.3	18.7	39.8	12.7	52.7
*p*-value*		<.0001	<.0001	0.219	<.0001	<.0001	<.0001
Education							
Low (0–2 years)	15.2	6.9	12	20.9	39.8	13.4	53.4
Low-avg (3–7 years)	58.3	7.1	14.5	18.2	43	13.7	56.5
High-avg (8–12 years)	17.4	18.3	34.3	20.3	59.1	23.1	73
High (13+ yrs)	9.1	19.2	36.8	21	59	25.2	73.6
*p*-value*		<.0001	<.0001	0.582	<.0001	<.0001	<.0001
Employment status							
Employed	49.7	7.2	16.5	19.2	42.6	15	58
Unemployed	30.8	12	19.3	19.3	51.9	18.8	64.1
Homemaker	9.8	34.2	55.5	22.4	66.6	31.1	86.4
Retired	2.6	31.5	57.1	28.2	65.3	26.6	81.5
Other	7.1	14.7	20.2	20.7	42.2	14.8	56.6
*p*-value*		<.0001	<.0001	0.561	<.0001	<.0001	<.0001
Race							
White	9.4	12.4	18.9	20.6	36.6	17.4	56.6
Black	75	9.6	19.9	18.8	48.6	16.9	61
Colored	10.2	11.5	19.5	19.4	43.1	13.4	57.8
Indian	3.1	9.8	15.9	25.3	48.2	17	66.1
Other	2.3	9.3	15.8	13.6	34.9	14.9	49.2
*p*-value*		0.631	0.802	0.577	0.01	0.604	0.255
Type of PTE							
War	11.2	10.4	21.5	31.9	50	21.5	65.6
*p*-value*		0.795	0.382	<.0001	0.276	0.009	0.073
Physical violence	32.9	10.6	21.7	24.3	55.6	20.6	67.8
*p*-value*		0.36	0.029	<.0001	<.0001	0.0003	<.0001
Sexual violence	28.4	14.1	26.8	27.9	60.1	25	72.8
*p*-value*		<.0001	<.0001	<.0001	<.0001	<.0001	<.0001
Accident	6.3	7.9	22.3	28.5	56.2	28	69
*p*-value*		0.332	0.226	0.002	0.015	<.0001	0.012
Unexpected death	31.3	14.1	26.4	24.6	56	21.5	70
*p*-value*		<.0001	<.0001	<.0001	<.0001	0.0003	<.0001
Network trauma	11.3	19.2	35	22.2	65.6	26.4	79.2
*p*-value*		<.0001	<.0001	0.128	<.0001	<.0001	<.0001
Witness	23.3	10.7	20.7	24.5	53.6	22.1	64.7
*p*-value*		0.539	0.357	<.0001	0.0003	<.0001	0.015
Any trauma	66.8	11.6	22.4	22.8	52.3	19.5	66
*p*-value*		<.0001	<.0001	<.0001	<.0001	<.0001	<.0001

^a^Totals represent the column totals for each demographic sub-population

*P-values represent the chi-sq test for differences in the frequency of PTE exposure among groups reporting each physical condition vs. not reporting that physical condition

### Frequency of cumulative PTE exposure

See Table 2 for full results. Respondents reporting zero PTEs with the greatest frequency were mostly female, aged 18–29, never married, highly educated (13+ yrs), employed as a homemaker, and identified as ‘colored’. Conversely, groups reporting the highest frequency of 5+ PTEs were mostly male, aged 30–44, with low (0–1 years) education, and identified their employment status and race as “other”.

**Table 2:** Prevalence of cumulative Potentially Traumatic Events (PTEs) among sociodemographic groups (*n* = 4351)

	Cumulative lifetime PTEs (%)^a^							Any PTE		% with PTSD	
	0	1	2	3	4	5+	*p*-value	%	*p*-value	%	*p*-value
Total (n; %)	1309 (27.8 %)	1005 (23.1 %)	917 (21.6 %)	591 (14.3 %)	328 (8.1 %)	199 (5.1 %)	<.0001	3040 (72.2 %)	<.0001	91 (2.2 %)	<.0001
Sex											
Female	27.7	23.6	21.7	14.6	8.3	4	<.0001	52.6	0.098	2.5	0.327
Male	25.4	19.8	21.4	15.3	10.7	7.4		47.4		1.9	
Age											
18–29	31.1	23.5	19.6	14.3	6.9	4.7	0.092	68.9	0.008	1.4	0.168
30–44	25.6	21.8	22.8	15.3	8.5	6		74.4		2.4	
45–59	24.1	23.7	23.1	13.9	9.9	5.4		75.9		3.2	
60+	29.8	23.4	23.6	11.6	8.1	3.5		70.2		3.1	
Marital Status											
Currently married	26.1	22	23.1	14.2	9.2	5.4	0.009	73.9	0.013	2.4	0.001
Previously married	21	27.2	17.4	19.4	10.2	4.8		79		6.9	
Never Married	30.8	23.5	20.6	13.8	6.4	4.8		69.2		1.3	
Education											
Low (0–2 years)	23.2	23.8	23.4	15.2	8.6	5.8	0.459	76.8	0.069	3.2	0.164
Low-avg (3–7 years)	27.9	22.8	21.4	14.3	8.1	5.5		72.1		1.8	
High-avg (8–12 years)	30.3	21.7	23.1	13.2	7.4	4.4		69.7		2.5	
High (13+ yrs)	29.7	25.3	18.4	15.2	8.6	2.9		70.3		2.8	
Employment status											
Employed	23.2	21.3	23.5	16	10.1	5.9	0.492	76.8	0.246	2.4	0.173
Unemployed	21.7	23	22.1	16.3	9.4	7.6		78.3		2.2	
Homemaker	27.1	20.2	27.4	11.9	10.3	3.2		72.9		5.2	
Retired	25.6	25	24.6	13.5	4.2	6.9		74.4		6.5	
Other	17.6	26.9	17.5	19.3	9.2	9.5		82.4		1.7	
Race											
White	26.6	23.2	23.4	11.7	10.8	4.3	0.10	73.4	0.155	3.8	0.118
Black	27.1	22.7	22	15	7.7	5.5		72.9		2	
Colored	35	24.4	17.3	13	7.7	2.6		65		3.1	
Indian	24.3	26.6	28.9	10.9	4.7	4.5		75.7		0.2	
Other	26.1	25	12.9	12.3	15.2	8.5		73.9		2.9	

^a^Does not include the PTE 'life-threatening illness’

*P*-values represent the differences in trends in the cumulative number of lifetime PTEs among demographic groups

### Odds ratios of physical conditions after exposure to PTEs

See Table 3 for detailed results. Compared to those reporting no PTE exposure, respondents with any PTE exposure had increased odds of reporting all assessed physical conditions, ranging between 1.48 (95 % CI: 1.06–2.07) for arthritis and 2.07 (95 % CI: 1.57–2.73) for respiratory conditions, after adjusting for sex, age, education, employment, marital status, race/ethnicity, and any mood, anxiety, PTSD, or substance use disorder.

**Table 3:** Multivariable odds ratios between each type of PTE and previous year physical conditions (*n* = 4351)

	Type of physical condition (OR, 95 % CI)					
PTE Type	Arthritis	Cardio-vascular	Respiratory	Chronic pain	Other Condition	Any physical condition
None	Ref	Ref	Ref	Ref	Ref	Ref
War	1.33	1.38	2.27	1.40	1.27	1.37
	(0.86–2.05)	(0.95–2.0)	(1.55–3.31)	(0.97–2.02)	(0.82–1.96)	(0.96–1.95)
Physical violence	1.32	1.56	1.50	1.69	1.73	1.61
	(0.99–1.76)	(1.25–1.96)	(1.18–1.89)	(1.39–2.07)	(1.34–2.24)	(1.25–2.08)
Sexual violence	1.56	1.52	1.76	2.06	1.71	1.97
	(1.19–2.05)	(1.12–2.04)	(1.36–2.29)	(1.64–2.58)	(1.35–2.17)	(1.57–2.48)
Accident	0.97	1.06	1.58	1.31	2.08	1.29
	(0.56–1.71)	(0.66–1.7)	(1.03–2.43)	(0.76–2.25)	(1.44–3.0)	(0.73–2.27)
Unexpected death	1.49	1.57	1.56	1.49	1.40	1.51
	(1.14–1.94)	(1.22–2.02)	(1.25–1.94)	(1.22–1.81)	(1.04–1.89)	(1.2–1.9)
Network trauma	1.71	1.84	1.18	2.27	1.89	2.48
	(1.16–2.53)	(1.36–2.5)	(0.86–1.61)	(1.7–3.04)	(1.3–2.75)	(1.72–3.58)
Witness	1.09	1.33	1.62	1.60	1.57	1.41
	(0.72–1.64)	(0.98–1.8)	(1.28–2.07)	(1.2–2.14)	(1.13–2.19)	(1.02–1.93)
Any trauma	1.48	1.71	2.07	1.74	1.76	1.67
	(1.06–2.07)	(1.22–2.41)	(1.57–2.73)	(1.37–2.21)	(1.32–2.36)	(1.34–2.09)

Model is adjusted for sex, age, education, employment, marital status, race/ethnicity, and any mood, anxiety, PTSD, or substance use disorder

Similarly, the odds of reporting any type of physical condition were significantly higher for individuals who reported experiencing all types of PTEs except war events or accidents; odds ranged between 1.41 (95 % CI: 1.02–1.93) among those who witnessed violence to 2.48 (95 % CI: 1.72–3.58) among those who experienced network events involving others. The odds of reporting any of the physical conditions were significantly elevated among individuals who experienced sexual violence or unexpected death of a loved one.

### Odds ratios of physical conditions by accumulated lifetime PTEs

See Table 4 for detailed results. Compared to individuals who reported no lifetime PTE exposure, the odds of physical conditions generally increased with each accumulated PTE. Individuals who reported one PTE exposure had increased odds for respiratory conditions; 62 % higher than the reference (95 % CI: 1.17–2.25). Among individuals with two or more PTEs, the odds of cardiovascular, respiratory, chronic pain, other health conditions, or any physical health condition were significantly higher. Among those with 4 or more PTEs, the odds of reporting arthritis, or any of the other physical conditions were significantly higher than for those without PTE exposure.

**Table 4:** Multivariable odds ratios between the number of lifetime PTEs and previous year physical conditions (*n* = 4351)

	Type of physical condition (OR, 95 % CI)					
Cumulative lifetime PTEs	Arthritis	Cardio-vascular	Respiratory	Chronic pain	Other	Any physical condition
0	Ref	Ref	Ref	Ref	Ref	Ref
1	1.12	1.27	1.62	1.06	0.96	1.10
	(0.71–1.77)	(0.86–1.89)	(1.17–2.25)	(0.77–1.45)	(0.65–1.4)	(0.84–1.44)
2	1.26	1.99	2.53	1.59	1.51	1.68
	(0.82–1.93)	(1.35–2.92)	(1.73–3.69)	(1.15–2.2)	(1.06–2.17)	(1.26–2.24)
3	0.95	1.469	2.19	1.95	2.30	1.98
	(0.63–1.42)	(0.91–2.36)	(1.54–3.11)	(1.4–2.72)	(1.63–3.25)	(1.4–2.79)
4	2.99	2.49	3.96	3.59	3.05	3.76
	(1.86–4.8)	(1.67–3.71)	(2.57–6.11)	(2.41–5.35)	(1.91–4.85)	(2.48–5.7)
5+	3.17	4.0	4.60	4.8	3.04	3.5
	(1.75–5.72)	(2.25–7.11)	(2.7–7.84)	(2.97–7.71)	(1.76–5.27)	(2.17–5.67)

Model is adjusted for sex, age, education, employment, marital status, race/ethnicity, and any mood, anxiety, PTSD, or substance use disorder

### Trends in prevalence of physical conditions by accumulated lifetime PTEs

**Fig. 1:**
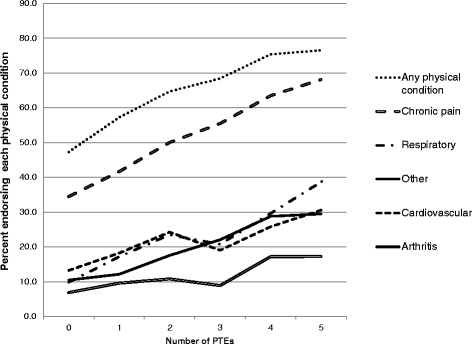
Percentage of study sample reporting physical conditions by the number cumulative PTEs reported

As shown in Fig. 1, there was an overall trend for an increase in the prevalence of all physical conditions with an increase in the number of reported lifetime PTEs. This trend is best illustrated with chronic pain, though it was statistically significant (*p* < .0001) for all physical conditions. A similar trend was seen in the association between cumulative number of PTEs and cumulative number of physical conditions (data available upon request).

## Discussion

This study found consistent evidence of associations between PTE exposures and increased risk for a wide range of chronic physical health conditions. These associations were independent of PTSD, other mental disorders, and sociodemographic factors. With respect to cumulative lifetime PTEs, findings suggest a linear dose-response association between number of PTEs experienced and prevalence of all physical conditions. This trend was most prominent with chronic pain, which was also the most prevalent physical condition. The dose-response relationship observed between multiple PTE exposures and increasing risk of a chronic physical conditions is well established in population-based [1, 19, 36, 37, 41] and other studies (eg [17, 21]).

This study is the first to examine the association between PTE exposure and chronic physical conditions in an African general population sample. Among other population-based studies controlling for mental disorders including PTSD, a 14-country cross-national study, and a U.S-based national sample, both reported increasing risk for a range of chronic physical health conditions with greater exposures to lifetime PTEs [1, 36]. However, in another study from the U.S, with a predominately African American sample, from Detroit, Michigan, Keyes and colleagues [19] reported a dose-dependent positive relationship only in relation to assaultive violence or other threats to physical integrity and risk of an arthritic condition. The study did not find similar associations for other types of PTEs or other health conditions.

Our study also found an increased risk of comorbidity in physical conditions with increasing numbers of PTEs. Specifically, those who reported only one lifetime PTE had a significantly higher risk for respiratory conditions only. Among individuals with two or more PTEs, the odds of having cardiovascular, respiratory, chronic pain, other problems, or any physical health condition, were significantly higher than those without PTE exposure. Finally, exposure to four or more PTEs was associated with increased risk of all the physical conditions examined. Our findings are consistent with studies suggesting “broad-spectrum effects” of PTEs and risk for a range of chronic physical problems [1, 41].

Compared to those individuals reporting no PTE exposure, respondents with any PTE exposure had increased odds of all assessed physical conditions. However, specific PTE types-sexual violence, physical violence, unexpected death of a loved one, and network PTEs-were associated with greater odds for most of the physical conditions assessed. While Keyes and colleagues (2013) found specific effects for assaultive violence or other threats to physical integrity only, our findings are more consistent with other studies reporting physical health problems following exposure to a range of PTEs [1].

This study also found that the odds of any type of physical condition were significantly higher for individuals experiencing any type of PTE except accidents or war events. It is possible that accidents may not show the same association with health outcomes because they maybe more likely to be acute single-incident stressors requiring short term biological adaptations, relative to other PTEs that are often repeated chronic stressors, which are associated with more biological wear and tear [52, 53]. With respect to war events, two other population-based studies [1, 36] have reported a similar lack of association with physical health problems. As previously noted in the literature, this finding may reflect the “healthy warrior” effect ie a sampling bias because individuals selected for deployment are medically screened and represent a healthier subset of a larger sample [1, 36, 54, 55], or they may be more likely to receive ongoing preventive medical attention [1, 55]. Another possibility is that the type of war-related PTE may moderate health outcomes; war veterans without direct combat exposure may not be at increased risk for certain health problems relative to those with combat exposure [56]. In our study, war exposure included a broad range of experiences (ie direct combat exposure, relief work or civilian presence in a war zone, refugee and purposely injured, and torturing or killing someone else). Thus, it is possible that certain types of war-related experiences may show different associations with physical conditions. Finally, it is plausible that PTE exposure during developmentally critical or sensitive periods in childhood may influence later health outcomes differently [57], and future studies should examine how timing of PTE exposure at various developmental stages influences chronic health problems.

Extant literature provides several possibilities for explanatory mechanisms for the associations between PTE exposures and chronic physical health outcomes. One possibility is that experience-dependent changes, in the context of chronic stressors such as PTEs may lead to abnormalities in multiple interlinked biological systems (eg, cortisol response, neurocognitive structure and function, immune functioning), which may underlie physical health outcomes [9]. Additionally, common posttraumatic reactions or behavioral coping mechanisms such as sleep problems [58], smoking [21, 59], or physical inactivity [28] may also underlie the observed associations. Finally, certain psychological coping styles (eg, emotional suppression) following PTEs may also influence physical health outcomes [9].

This study has important limitations. First, we relied on retrospective self-reports of PTE exposures, a method that is likely to underreport most events. Our use of a detailed PTE checklist may have mitigated the likelihood of under-reporting, but reporting bias cannot be completely eliminated [7]. Future studies should consider multi-method assessments for PTE exposures (eg, clinician interviews). Second, physical health conditions were also based on self-report. While self-reports have often shown concordance with physician or medical record diagnoses [60, 61], the possibility of misclassification cannot be ruled out. Finally, the cross-sectional observational nature of our study precludes causal attributions. However, our findings are in line with related longitudinal studies [62] which provide strong evidence associations between PTE and physical health outcomes. Future studies should build on this and related clinical guidelines [63, 64], and seek to evaluate the feasibility and utility of trauma-informed screenings/intervention in primary care.

## Conclusion

This study adds to an increasing body of research suggesting that PTE exposure is not only a risk factor for mental disorders, but also for physical health conditions. Our findings also show that associations between PTEs and physical health conditions are not fully mediated by psychiatric disorders including common PTE sequelae (eg, PTSD). We found a dose-response association between cumulative exposure to PTEs and the odds of reporting chronic physical health conditions and believe that clinical evaluations of trauma survivors should consider assessments of mental and physical health.

## References

[1] Scott KM, Koenen KC, Aguilar-Gaxiola S, Alonso J, Angermeyer MC, Benjet C, Bruffaerts R, Caldas-de-Almeida JM, de Girolamo G, Florescu S (2013). Associations between lifetime traumatic events and subsequent chronic physical conditions: a cross-national, cross-sectional study. PLoS One.

[2] Gilbert LK, Breiding MJ, Merrick MT, Thompson WW, Ford DC, Dhingra SS, Parks SE (2014). Childhood adversity and adult chronic disease: an update from Ten states and the district of Columbia, 2010. Am J Prev Med.

[3] Spitzer C, Barnow S, Volzke H, John U, Freyberger HJ, Grabe HJ (2009). Trauma, posttraumatic stress disorder, and physical illness: findings from the general population. Psychosom Med.

[4] Qureshi SU, Pyne JM, Magruder KM, Schulz PE, Kunik ME (2009). The link between post-traumatic stress disorder and physical comorbidities: a systematic review. Psychiatry Q.

[5] Wegman HL, Stetler C (2009). A meta-analytic review of the effects of childhood abuse on medical outcomes in adulthood. Psychosom Med.

[6] Paolucci EO, Genuis ML, Violato C (2001). A meta-analysis of the published research on the effects of child sexual abuse. J Psychol.

[7] Atwoli L, Stein DJ, Williams DR, McLaughlin KA, Petukhova M, Kessler RC, Koenen KC (2013). Trauma and posttraumatic stress disorder in South Africa: analysis from the South African Stress and Health Study. BMC Psychiatry.

[8] Unwin N (2001). Non-communicable disease and priorities for health policy in sub-Saharan Africa. Health Policy Plan.

[9] D'Andrea W, Sharma R, Zelechoski AD, Spinazzola J (2011). Physical health problems after single trauma exposure: when stress takes root in the body. J Am Psychiatr Nurses Assoc.

[10] Suglia SF, Sapra KJ, Koenen KC (2015). Violence and cardiovascular health: a systematic review. Am J Prev Med.

[11] Boscarino JA (2012). PTSD is a risk factor for cardiovascular disease: time for increased screening and clinical intervention. Prev Med.

[12] Vaccarino V, Goldberg J, Rooks C, Shah AJ, Veledar E, Faber TL, Votaw JR, Forsberg CW, Bremner JD (2013). Post-traumatic stress disorder and incidence of coronary heart disease: a twin study. J Am Coll Cardiol.

[13] Davis DA, Luecken LJ, Zautra AJ (2005). Are reports of childhood abuse related to the experience of chronic pain in adulthood? A meta-analytic review of the literature. Clin J Pain.

[14] Lang AJ, Aarons GA, Gearity J, Laffaye C, Satz L, Dresselhaus TR, Stein MB (2008). Direct and indirect links between childhood maltreatment, posttraumatic stress disorder, and women's health. Behav Med.

[15] Lang AJ, Laffaye C, Satz LE, McQuaid JR, Malcarne VL, Dresselhaus TR, Stein MB (2006). Relationships among childhood maltreatment, PTSD, and health in female veterans in primary care. Child Abuse Negl.

[16] Kimerling R, Clum GA, Wolfe J (2000). Relationships among trauma exposure, chronic posttraumatic stress disorder symptoms, and self-reported health in women: replication and extension. J Trauma Stress.

[17] Anda RF, Tietjen G, Schulman E, Felitti V, Croft J (2010). Adverse childhood experiences and frequent headaches in adults. Headache.

[18] Dube SR, Fairweather D, Pearson WS, Felitti VJ, Anda RF, Croft JB (2009). Cumulative childhood stress and autoimmune diseases in adults. Psychosom Med.

[19] Keyes KM, McLaughlin KA, Demmer RT, Cerda M, Koenen KC, Uddin M, Galea S (2013). Potentially traumatic events and the risk of six physical health conditions in a population-based sample. Depress Anxiety.

[20] Boscarino JA, Forsberg CW, Goldberg J (2010). A twin study of the association between PTSD symptoms and rheumatoid arthritis. Psychosom Med.

[21] Anda RF, Brown DW, Dube SR, Bremner JD, Felitti VJ, Giles WH (2008). Adverse childhood experiences and chronic obstructive pulmonary disease in adults. Am J Prev Med.

[22] Bhan N, Glymour MM, Kawachi I, Subramanian SV (2014). Childhood adversity and asthma prevalence: evidence from 10 US states (2009–2011). BMJ Open Respir Res.

[23] Rich-Edwards JW, Spiegelman D, Lividoti Hibert EN, Jun HJ, Todd TJ, Kawachi I, Wright RJ (2010). Abuse in childhood and adolescence as a predictor of type 2 diabetes in adult women. Am J Prev Med.

[24] Mason SM, Wright RJ, Hibert EN, Spiegelman D, Jun HJ, Hu FB, Rich-Edwards JW (2013). Intimate partner violence and incidence of type 2 diabetes in women. Diabetes Care.

[25] Roberts AL, Agnew-Blais JC, Spiegelman D, Kubzansky LD, Mason SM, Galea S, Hu FB, Rich-Edwards JW, Koenen KC (2015). Posttraumatic Stress Disorder and Incidence of Type 2 Diabetes Mellitus in a Sample of Women: A 22-Year Longitudinal Study. JAMA Psychiatry.

[26] Norman SB, Means-Christensen AJ, Craske MG, Sherbourne CD, Roy-Byrne PP, Stein MB (2006). Associations between psychological trauma and physical illness in primary care. J Trauma Stress.

[27] Boscarino JA (2008). Psychobiologic predictors of disease mortality after psychological trauma: implications for research and clinical surveillance. J Nerv Ment Dis.

[28] Felitti VJ, Anda RF, Nordenberg D, Williamson DF, Spitz AM, Edwards V, Koss MP, Marks JS (1998). Relationship of childhood abuse and household dysfunction to many of the leading causes of death in adults. The Adverse Childhood Experiences (ACE) Study. Am J Prev Med.

[29] Clark CJ, Everson-Rose SA, Alonso A, Spencer RA, Brady SS, Resnick MD, Borowsky IW, Connett JE, Krueger RF, Suglia SF (2014). Effect of partner violence in adolescence and young adulthood on blood pressure and incident hypertension. PLoS One.

[30] Mason SM, Wright RJ, Hibert EN, Spiegelman D, Forman JP, Rich-Edwards JW (2012). Intimate partner violence and incidence of hypertension in women. Ann Epidemiol.

[31] Rich-Edwards JW, Mason S, Rexrode K, Spiegelman D, Hibert E, Kawachi I, Jun HJ, Wright RJ (2012). Physical and sexual abuse in childhood as predictors of early-onset cardiovascular events in women. Circulation.

[32] Irish LA, Gabert-Quillen CA, Ciesla JA, Pacella ML, Sledjeski EM, Delahanty DL (2013). An examination of PTSD symptoms as a mediator of the relationship between trauma history characteristics and physical health following a motor vehicle accident. Depress Anxiety.

[33] O'Toole BI, Catts SV (2008). Trauma, PTSD, and physical health: an epidemiological study of Australian Vietnam veterans. J Psychosom Res.

[34] McFarlane AC (2010). The long-term costs of traumatic stress: intertwined physical and psychological consequences. World Psychiatry.

[35] Vedantham K, Brunet A, Boyer R, Weiss DS, Metzler TJ, Marmar CR (2001). Posttraumatic stress disorder, trauma exposure, and the current health of Canadian bus drivers. Can J Psychiatry.

[36] Husarewycz MN, El-Gabalawy R, Logsetty S, Sareen J (2014). The association between number and type of traumatic life experiences and physical conditions in a nationally representative sample. Gen Hosp Psychiatry.

[37] Sledjeski EM, Speisman B, Dierker LC (2008). Does number of lifetime traumas explain the relationship between PTSD and chronic medical conditions? Answers from the National Comorbidity Survey-Replication (NCS-R). J Behav Med.

[38] Pietrzak RH, Goldstein RB, Southwick SM, Grant BF (2012). Physical health conditions associated with posttraumatic stress disorder in U.S. older adults: results from wave 2 of the National Epidemiologic Survey on Alcohol and Related Conditions. J Am Geriatr Soc.

[39] Pietrzak RH, Goldstein RB, Southwick SM, Grant BF (2011). Medical comorbidity of full and partial posttraumatic stress disorder in US adults: results from Wave 2 of the National Epidemiologic Survey on Alcohol and Related Conditions. Psychosom Med.

[40] Kessler RC, Rose S, Koenen KC, Karam EG, Stang PE, Stein DJ, Heeringa SG, Hill ED, Liberzon I, McLaughlin KA (2014). How well can post-traumatic stress disorder be predicted from pre-trauma risk factors? An exploratory study in the WHO World Mental Health Surveys. World Psychiatry.

[41] Scott KM, Von Korff M, Angermeyer MC, Benjet C, Bruffaerts R, de Girolamo G, Haro JM, Lepine JP, Ormel J, Posada-Villa J (2011). Association of childhood adversities and early-onset mental disorders with adult-onset chronic physical conditions. Arch Gen Psychiatry.

[42] Williams DR, Herman A, Kessler RC, Sonnega J, Seedat S, Stein DJ, Moomal H, Wilson CM (2004). The South Africa Stress and Health Study: rationale and design. Metab Brain Dis.

[43] Herman AA, Stein DJ, Seedat S, Heeringa SG, Moomal H, Williams DR (2009). The South African Stress and Health (SASH) study: 12-month and lifetime prevalence of common mental disorders. S Afr Med J.

[44] American Psychiatric Association (1994). Diagnostic and statistical manual of mental disorders.

[45] Kessler RC, Ustun TB (2004). The World Mental Health (WMH) Survey Initiative Version of the World Health Organization (WHO) Composite International Diagnostic Interview (CIDI). Int J Methods Psychiatr Res.

[46] Colditz GA, Martin P, Stampfer MJ, Willett WC, Sampson L, Rosner B, Hennekens CH, Speizer FE (1986). Validation of questionnaire information on risk factors and disease outcomes in a prospective cohort study of women. Am J Epidemiol.

[47] Bush TL, Miller SR, Golden AL, Hale WE (1989). Self-report and medical record report agreement of selected medical conditions in the elderly. Am J Public Health.

[48] Smith B, Chu LK, Smith TC, Amoroso PJ, Boyko EJ, Hooper TI, Gackstetter GD, Ryan MA (2008). Challenges of self-reported medical conditions and electronic medical records among members of a large military cohort. BMC Med Res Methodol.

[49] Khalfani AK, Zuberi T (2001). Racial classification and the modern census in South Africa, 1911–1996. Race Soc.

[50] Margolin B (1988). Test for trend in proportions. Encycloped Stat Sci.

[51] StataCorp (2011). Stata Statistical Software: Release 12.

[52] McEwen BS (2004). Protection and damage from acute and chronic stress: allostasis and allostatic overload and relevance to the pathophysiology of psychiatric disorders. Ann N Y Acad Sci.

[53] Dhabhar FS (2008). Enhancing versus Suppressive Effects of Stress on Immune Function: Implications for Immunoprotection versus Immunopathology. Allergy Asthma Clin Immunol.

[54] Haley RW (1998). Point: bias from the "healthy-warrior effect" and unequal follow-up in three government studies of health effects of the Gulf War. Am J Epidemiol.

[55] Tansey CM, Raina P, Wolfson C (2012). Veterans' physical health. Epidemiol Rev.

[56] Johnson AM, Rose KM, Elder GH, Chambless LE, Kaufman JS, Heiss G (2010). Military combat and risk of coronary heart disease and ischemic stroke in aging men: The Atherosclerosis Risk in Communities (ARIC) study. Ann Epidemiol.

[57] Slopen N, McLaughlin KA, Dunn EC, Koenen KC (2013). Childhood adversity and cell-mediated immunity in young adulthood: does type and timing matter?. Brain Behav Immun.

[58] Babson KA, Feldner MT (2010). Temporal relations between sleep problems and both traumatic event exposure and PTSD: a critical review of the empirical literature. J Anxiety Disord.

[59] Dong M, Giles WH, Felitti VJ, Dube SR, Williams JE, Chapman DP, Anda RF (2004). Insights into causal pathways for ischemic heart disease: adverse childhood experiences study. Circulation.

[60] Baumeister H, Kriston L, Bengel J, Harter M (2010). High agreement of self-report and physician-diagnosed somatic conditions yields limited bias in examining mental-physical comorbidity. J Clin Epidemiol.

[61] Kriegsman DM, Penninx BW, van Eijk JT, Boeke AJ, Deeg DJ (1996). Self-reports and general practitioner information on the presence of chronic diseases in community dwelling elderly. A study on the accuracy of patients' self-reports and on determinants of inaccuracy. J Clin Epidemiol.

[62] Sumner JA, Kubzansky LD, Kabrhel C, Roberts AL, Chen Q, Winning A, Gilsanz P, Rimm EB, Glymour MM, Koenen KC (2016). Associations of trauma exposure and posttraumatic stress symptoms with venous thromboembolism over 22 years in women. J Am Heart Assoc.

[63] Raja S, Hasnain M, Hoersch M, Gove-Yin S, Rajagopalan C (2015). Trauma informed care in medicine: current knowledge and future research directions. Fam Community Health.

[64] Ursano RJ, Benedek DM, Engel CC (2012). Trauma-informed care for primary care: the lessons of war. Ann Intern Med.

